# Multicolor spectral photon counting CT monitors and quantifies therapeutic cells and their encapsulating scaffold in a model of brain damage

**DOI:** 10.7150/ntno.45354

**Published:** 2020-04-22

**Authors:** Elisa Cuccione, Peter Chhour, Salim Si-Mohamed, Chloé Dumot, Johoon Kim, Violaine Hubert, Claire Crola Da Silva, Marc Vandamme, Emmanuel Chereul, Joëlle Balegamire, Yves Chevalier, Yves Berthezène, Loïc Boussel, Philippe Douek, David P. Cormode, Marlène Wiart

**Affiliations:** 1 CarMeN Laboratory, Institut National de la Santé et de la Recherche Médicale U1060, INRA U1397, Université Lyon 1, INSA Lyon, F-69600 Oullins, France.; 2 Department of Radiology, University of Pennsylvania, Pennsylvania, United States; 3 CREATIS, CNRS UMR 5220 - INSERM U1206 - University of Lyon 1 - INSA Lyon, Lyon, France; 4 Hospices Civils de Lyon, Radiology Department, Lyon, France; 5 VOXCAN, 1 avenue Bourgelat, 69280 Marcy l'Etoile, France; 6 LAGEPP, University of Lyon 1, CNRS UMR 5007, 43 bd 11 Novembre, 69622 Villeurbanne, France

**Keywords:** multicolor spectral photon-counting CT, regenerative medicine, cell therapy, cell tracking, neurology

## Abstract

**Rationale & aim**: Various types of cell therapies are currently under investigation for the treatment of ischemic stroke patients. To bridge the gap between cell administration and therapeutic outcome, there is a need for non-invasive monitoring of these innovative therapeutic approaches. Spectral photon counting computed tomography (SPCCT) is a new imaging modality that may be suitable for cell tracking. SPCCT is the next generation of clinical CT that allows the selective visualization and quantification of multiple contrast agents. The aims of this study are: (i) to demonstrate the feasibility of using SPCCT to longitudinally monitor and quantify therapeutic cells, i.e. bone marrow-derived M2-polarized macrophages transplanted in rats with brain damage; and (ii) to evaluate the potential of this approach to discriminate M2-polarized macrophages from their encapsulating scaffold.

**Methods**: Twenty one rats received an intralesional transplantation of bone marrow-derived M2-polarized macrophages. In the first set of experiments, cells were labeled with gold nanoparticles and tracked for up to two weeks post-injection in a monocolor study via gold K-edge imaging. In the second set of experiments, the same protocol was repeated for a bicolor study, in which the labeled cells are embedded in iodine nanoparticle-labeled scaffold. The amount of gold in the brain was longitudinally quantified using gold K-edge images reconstructed from SPCCT acquisition. Animals were sacrificed at different time points post-injection, and ICP-OES was used to validate the accuracy of gold quantification from SPCCT imaging.

**Results**: The feasibility of therapeutic cell tracking was successfully demonstrated in brain-damaged rats with SPCCT imaging. The imaging modality enabled cell monitoring for up to 2 weeks post-injection, in a specific and quantitative manner. Differentiation of labeled cells and their embedding scaffold was also feasible with SPCCT imaging, with a detection limit as low as 5,000 cells in a voxel of 250 × 250 × 250 µm in dimension *in vivo*.

**Conclusion**: Multicolor SPCCT is an innovative translational imaging tool that allows monitoring and quantification of therapeutic cells and their encapsulating scaffold transplanted in the damaged rat brain.

## Introduction

Stroke is the leading cause of long-term disability with no effective cure after the first hours of onset. Various types of cell therapies are currently being evaluated in many clinical trials involving patients with ischemic stroke 1. A meta-analysis of preclinical studies found that the intracerebral administration is associated with the best treatment efficacy compared to other administration routes, as they can thus act locally by secreting neurotrophic factors for example 2-4. Recently, two independent clinical trials reported the safety of intracerebral administration of different types of therapeutic cells in stroke patients 5, 6. The infarct cavity allows the injection of a large volume of cells; however, it is a hostile environment for cell engraftment. To improve the integration of the grafted cells, these cells can be embedded within a bioengineered scaffold 7. To bridge the gap between the administration of regenerative therapy and the clinical trial outcome, the regulatory agencies that approve advanced-therapy medicinal products (ATMPs) have highlighted the need for non-invasive imaging tools 8. The challenge is to propose a solution that involves clinically relevant imaging techniques, in order to foster the clinical translation of these innovative theranostic approaches.

In human trials of cell tracking, nuclear medicine imaging, such as positron emission tomography (PET) and single photon emission computed tomography (SPECT), is mainly used 9. However, the relatively short half-lives of radiotracers prevent the long-term tracking of administrated cells. Gene reporter imaging with PET or SPECT may overcome this problem 10, although to date these technologies remain costly and not widely available. Magnetic resonance imaging (MRI) and X-ray computed tomography (CT) are the leading three-dimensional (3D) radiological technologies around the world. Coupled with contrast agents, both modalities are regarded as the imaging techniques that can potentially track administered cells up to several weeks. To render cells detectable, a contrast agent is incorporated into cells prior to transplantation. Superparamagnetic particles of iron oxide (SPIO) and gold nanoparticles (AuNPs) are examples of widely studied contrast agents for MRI and CT, respectively 11, 12. One limitation of both these approaches is the lack of specificity of the signal produced by the labeled cells, which makes it difficult to non-equivocally distinguish the signal of labeled cells from other sources of hyperdensities (microhemorrhages or calcifications for instance). A recent breakthrough in CT technology has been the introduction of dual-energy CT (DECT) into clinical CT scanners. DECT has several advantages compared to conventional CT, including better contrast-to-noise ratio and fewer artifacts 13. Importantly, after the administration of an iodinated contrast agent, DECT allows the reconstruction of non-contrast and contrast-enhanced images based on a single acquisition, eliminating the need for pre-scan. This leads to X-ray dose reduction without altering the diagnostic value. The development of spectral photon-counting detectors rather than energy-integrating detectors has extended the DECT approach to a multispectral approach. This next generation spectral photon counting CT (SPCCT) is expected to further expand the boundaries of spectral CT in terms of noise reduction and tissue differentiation 14. It also improves the intrinsic spatial resolution owing to small pixel detector size (~250 µm) 15, 16. In addition, SPCCT allows selective visualization and quantification of multiple contrast agents by exploiting the K-edge discontinuity in X-ray absorption, a concept coined 'K-edge imaging' or 'multicolor CT' 17. The first reports of bicolor imaging with SPCCT have demonstrated the potential of this innovative approach 18-23, with many future applications yet to be developed.

This study aims to: (i) demonstrate the feasibility of using SPCCT to longitudinally monitor and quantify AuNP-labeled therapeutic cells transplanted into brain-damaged rats; and (ii) evaluate the potential of this approach to discriminate the labeled cells from their encapsulating scaffold, both labeled with different types of contrast agents. Bone marrow-derived M2-polarized (repair) macrophages were used as the therapeutic cells 24, 25. The commercially-available scaffold PuraMatrix® was employed to encapsulate the cells 26, 27. Stereotaxic injection into rodent brains was chosen as a mean to model intracerebral administration in humans. A SPCCT prototype system (Philips Healthcare, Haifa, Israel) with small field of view (16.8 cm) was used for image acquisition 28. This system has five thresholds that could be adjusted in order to allow photon energy-based discrimination of any given element that has a K-edge in the range of 30 - 120 keV.

## Materials and Methods

### Animals

All experimental procedures involving animals and their care were carried out in accordance with the European regulation for animal use (APAFIS agreement number #4688). This study was approved by the local ethics committee of our institution (C2EA - 42, local ethic board). Adult male Sprague-Dawley rats (Janvier, France) that were 6 to 7 weeks-old at reception and with a body weight in the range of 250-300 g and C57Bl/6 mice (Janvier, France) that were 5 weeks-old at reception and with a body weight in the range of 25-30 g were used. An acclimation period of at least 7 days was respected. Animals were housed in a temperature- and humidity-controlled environment (21 ± 3 °C), on 12:12 h light-dark cycle, having free access to standard chow and tap water. Housing Pexiglas cages were bedded with wood dust and enriched with tunnels and wood sticks (rats) or domes (mice). The data are reported according to the ARRIVE guidelines (Animal Research: Reporting of *In vivo* Experiments).

### Contrast agents

#### Gold nanoparticles (AuNPs)

11-Mercaptoundecanoic acid capped gold nanoparticles (11-MUDA AuNPs) were synthesized via a previously reported adaptation of the Turkevich method 29, 30. In brief, 85 mg of gold (III) chloride salt was dissolved in 500 ml of ultrapure water and brought to a boil while stirring. 25 mL of sodium citrate (38.8 mM) was added and the solution allowed to boil for additional 15 minutes before cooling to room temperature. A wine-red solution of gold nanoparticles resulted from this procedure. To cap the gold nanoparticles, 2.6 mg of 11-MUDA dissolved in 1 mL of ethanol was added, and the solution was stirred overnight. The resulting 11-MUDA AuNPs were purified by centrifuging them three times at 8.5 krcf and exchanging the supernatant with ultrapure water each time. AuNPs were then sterilized via syringe filtration (size: 0.45 µm) before further use. These nanoparticles had the following characteristics: peak absorbance of 524 nm, average hydrodynamic diameter of 22 nm with PDI of 0.2, core size of 11 ± 1 nm, and zeta potential of -44.4 mV.

#### Iodinated nanoparticles (INPs)

Concentrated aqueous suspensions of INPs were prepared in two steps of emulsification and concentration as follows 31. The iodinated polymer TIB-PVAL was a 2,3,5-triodobenzoyl ester of poly(vinyl alcohol) containing 70 wt% of iodine. The emulsification of TIB-PVAL in water was performed by mixing 25 mL of 4 wt% TIB-PVAL in THF and 50 mL of deionized water. A block copolymer polycaprolactone-*block*-poly(ethylene glycol) was used as an emulsifier. After evaporation of THF under reduced pressure, the resulting aqueous suspension of INPs had a concentration of 15 mg iodine/mL. As the second step, the iodine concentration was increased up to 100 mg(I)/mL by means of centrifugation and redispersion of the pellet into a small volume of water. INPs had the following characteristics: peak absorbance of 237 nm, hydrodynamic diameter of 122 nm with PDI of 0.2, and core size of 100 ± 15 nm.

### Cell culture and cell labeling

Bone marrow cells were harvested by flushing out tibias and femurs of donor animals. Total bone marrow cells were seeded on uncoated 6-well plates at 5×10^5^ cells/mL in RPMI 1640 with Glutamax (Fisher Scientific, Illkirch, France) supplemented with 10% fetal calf serum (SVF premium, Dutscher, France), 1% penicillin-streptomycin Fisher Scientific, Illkirch, France) and 25 ng/mL of mouse macrophage-colony stimulating factor (mM-CSF) (Miltenyi Biotec, Paris, France). The cell cultures were incubated at 37 °C in humidified 5% CO_2_. Seven days after plating, non-adherent cells were harvested and adherent cells were washed twice in phosphate-buffered saline (PBS). The harvested bone marrow-derived macrophages were then stimulated with interleukin-4 (20 ng/mL) (Miltenyi Biotec, Paris, France) for 48 h to generate M2 alternatively-activated macrophages. Macrophage cell culture purity under these experimental conditions was repeatedly found to exceed 95% on flow cytometry analysis of CD11b+ cells (data not shown). At day 6, cells were incubated with AuNPs at 0.1 mg/mL for 18 h, based on a protocol that provided high AuNPs uptake while maintaining cell viability 29. Immediately after labeling, cells were detached by trypsination, washed once in PBS, counted and resuspended in Ca^2+^-Mg^2+^ sterile PBS for *in vivo* injection. AuNP internalization and cell morphology post-labeling were assessed by light microscopy. The viability of the AuNP labeled cells was examined using the LIVE/DEAD assay (Invitrogen, Carlsbad, CA, USA). The efficiency of labeling was determined based on inductively coupled plasma-optical emission spectrometry (ICP-OES) using three different bone-marrows and triplicates for each bone-marrow.

### Scaffold and scaffold labeling

Puramatrix (3-D Matrix, MA, USA) is a synthetic peptide that undergoes self-assembly into nanofiber hydrogels similar to the extracellular matrix upon introduction of monovalent cations in physiological conditions. PuraMatrix thus provides a suitable biological scaffold for cell transplantation, and it has been used for central nervous system regeneration 27, 32. For injection of AuNPs-labeled cells in INPs-labeled scaffold, the total volume of injectable solution (10 µL) was composed of PuraMatrix (1/4 of 10 µL), INPs solution (1/8 of 10 µL) and AuNPs-labeled cells in PBS (remaining volume).

### *In vitro* studies

The accuracy of quantification in SPCCT material imaging has been demonstrated previously by phantom imaging of iodine, gold and their mixture with other contrast agents 17. In this study, thirteen samples were prepared by suspending the gold or iodine nanoparticles in 1% agarose gel placed in Eppendorf tubes with a range of concentrations: 0, 10, 15, 20, 30, 40 and 60 mM for INPs and 0, 10, 15, 20, 30, and 40 mM for AuNPs (thus resulting in the same range of 0 - 8 mg/mL for each material). These phantoms were scanned at each imaging time point for calibration purpose in longitudinal *in vivo* studies (see below). In addition, to evaluate the performance of SPCCT quantification in our experimental setting, labeled cells pellets were prepared in the same conditions as for the *in vivo* administration, i.e. 10 µL of PBS with decreasing quantity of cells: 1 x 10^6^, 0.5 x 10^6^, 0.25 x 10^6^, 0.125 x 10^6^ and no cells, placed at the bottom of Eppendorf tubes and secured with 1% agarose gel on top.

### *In vivo* studies

#### Overall protocol

**Figure 1:**
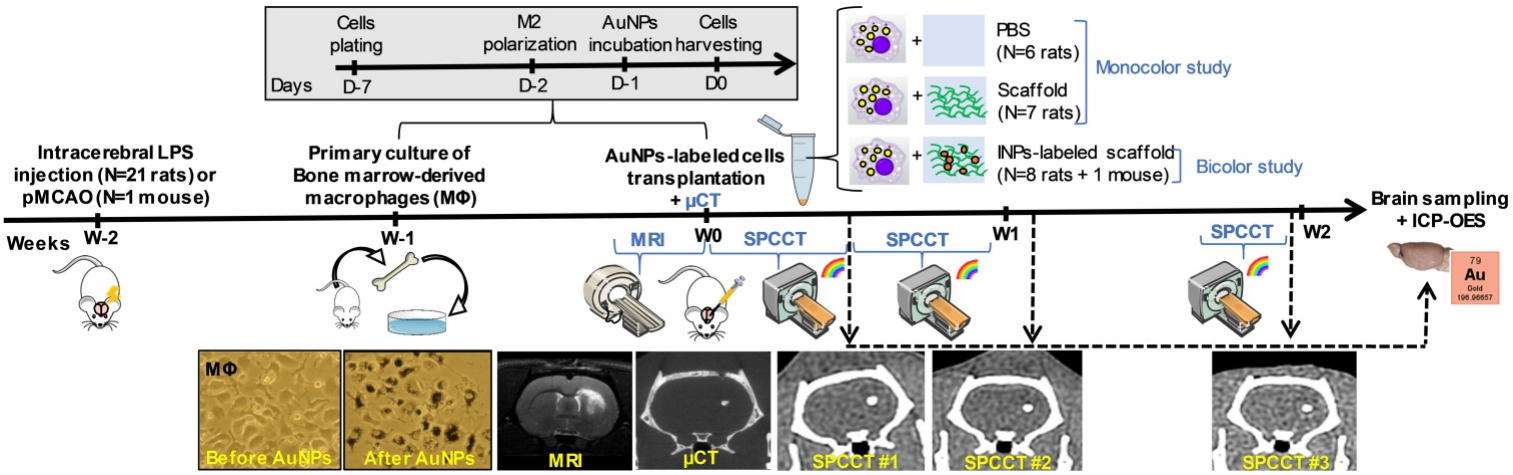
** Experimental timeline for longitudinal multimodal imaging of brain-damaged rats transplanted with cell therapy.** AuNPs: gold nanoparticles; ICP-OES: inductively coupled plasma-optical emission spectrometry; INPs: iodinated nanoparticles; LPS: lipopolysaccharide; µCT: micro-computed tomography; MRI: magnetic resonance imaging; pMCAO: permanent middle cerebral artery occlusion; SPCCT: spectral photon counting computed tomography.

Figure 1 shows the experimental design of *in vivo* studies. In order to generate a lesion cavity that mimics stroke infarct at the chronic stage, 21 rats (experimental units) received an intracerebral injection of lipopolysaccharide (LPS) 33. To evaluate the exact lesion location for cell administration, rat brains were imaged 2 weeks later by T_2_-weighted MRI. Subsequently, the rats received an intra-lesional injection of different therapeutic materials (e.g. cells ± scaffold) as detailed thereafter. For this set of experiments, rats were randomly assigned to injection with or without scaffold. In the first set of experiments, cells were labeled with gold nanoparticles (AuNPs) and tracked up to two weeks post-injection using K-edge imaging of gold (monocolor study, n=13 LPS-treated rats). For the second set of experiments, the same protocol was repeated for a bicolor study, in which the labeled cells are embedded in iodine nanoparticle (INP)-labeled scaffold (bicolor study, n=8 LPS-treated rats). In both cases, the success of transplantation was determined by performing µCT immediately after intracerebral cell administration. The µCT images are also shown as images of reference for accurate cell location. Sample size was determined in order to obtain enough data for this proof-of-concept study.

#### Animal models and cell transplantation

Animals were anesthetized by breathing 3-4% isoflurane (ISO-VET, Piramal Healthcare, Morpeth, UK) in air and maintained under 1.5% isoflurane in air using a face mask and mounted in a stereotaxic apparatus (D. Kopf Instruments). Isoflurane anesthesia was selected since it allows modulating anesthesia depth. Rectal temperature was kept at 37 ± 1 °C throughout the surgical procedures, using a feedback-regulated heating pad. To alleviate pain, buprenorphine was administered subcutaneously at the dose of 0.05 mg/kg prior to the surgery. Lubricant ophthalmic gel was applied on both eyes to prevent ocular dehydration, and the surgical site was shaved and disinfected with antiseptic solution (i.e. 10 % iodopovidone). To mimic stroke infarct, rats were stereotaxically injected with 50 µg of LPS from Escherichia coli (Sigma-Aldrich, Saint-Louis, USA) dissolved in 4 µL saline in the right hemisphere of the brain (0.5 mm anterior to bregma, 3.0 mm in lateral direction, 2 µL at 5.5 mm and 2 µL at 4.5 mm deep from the cortical surface). In addition, one mouse was submitted to ischemic stroke by permanent middle cerebral artery occlusion (pMCAO) according to a protocol published elsewhere 34. Briefly, the right distal middle cerebral artery was exposed by subtemporal craniotomy and occluded by electrocoagulation. AuNP-labeled cells (in the range of 0.125 x 10^6^ to 0.5 x 10^6^) were intracerebrally transplanted in 10 µL vehicle (PBS or scaffold) inside the lesion, using stereotaxic coordinates based on pre-surgery MRI for each animal. At the end of the surgery, the wounds were cleaned with sterile saline solution, disinfected, and covered with lidocaine. Animals were allowed to completely wake up from anesthesia under a heating lamp and then housed individually for approximately 24 hours after the surgery. The surgical wound appearance was visually checked for correct healing.

#### Monocolor study

Thirteen LPS-treated rats were intracerebrally administered with 0.5 x 10^6^ AuNP-labeled cells encapsulated either in PBS (n=6) or in scaffold (n=7). These rats were imaged with SPCCT three times during the first 2 weeks of post-transplantation period - once in the first half of the first week (Day 0-3), once in the second half of the first week (Day 4-7), and once at the end of the second week (Day 13-14). Subgroups of rats were sacrificed at each time point to compare the gold content estimated with SPCCT analysis with the gold content measured by ICP-OES (n=2 at Day 0-3, n=7 at Day 4-7 and n=4 at Day 13-14).

#### Bicolor study

To evaluate the feasibility of bicolor imaging, two LPS-treated rats and one pMCAO mouse were intracerebrally administered with 0.5 x 10^6^ cells encapsulated in 10 µL iodine-labeled scaffold and then imaged twice during the first week of post-administration (once in Day 1-2 and once in Day 4-6). Lastly, to evaluate the limits of detection for AuNP-labeled cells in the context of bicolor imaging, 6 rats received a range of cell quantities, i.e. 0.5 x 10^6^ (n=2), 0.25 x 10^6^ (n=2) or 0.125 x 10^6^ (n=2). The iodine quantity in the scaffold did not vary between groups. These rats were imaged twice during the first week (at Day 1-2 and Day 4-6) and once at the end of the second week (Day 12-14).

### *In vivo* multimodal imaging

All imaging procedures were performed under 1-2% isoflurane anesthesia. System characteristics and acquisition parameters for MRI, µCT, CT and SPCCT are presented in Table 1. The SPCCT scanner is described in details in previous studies 17, 28. In brief, it is based on a clinical iCT platform (Philips Healthcare, Haifa, Israel), operating with a standard X-ray source and energy-resolving photon counting detectors made of cadmium-zinc telluride, with 5 energy thresholds that can be tuned according to the materials of interest. Tube voltages can be set to 80, 100 or 120 kVp and tube currents from 10 to 100 mA. The effective z-collimation is 2.5 mm in the isocenter, in-plane field of view is 168 mm, Z-coverage is 2 mm and minimum rotation time is 0.75 sec/rotation. We acquired images using both axial and helical acquisition modes at a tube current of 100 mA and a tube voltage of 120 kVp with the energy thresholds of 30, 53, 78, 83 and 98 keV. For reconstruction, the image chain used a two-step material decomposition approach that first generates material specific sinograms for each material of interest (e.g. gold, iodine, gadolinium, water), which is followed by reconstruction of each sinogram into specific material image, depending on the user's choice. Taken together, the specific images are generated by best fit of the measured count rates in the five energy bins available to calibration data from phantom imaging. A maximum-likelihood based material decomposition 35 based on literature data of the attenuations 36 of the target materials was applied to derive material sinograms. Further details are provided in previous studies 17, 22.

### Image analysis

Image analysis was performed using ImageJ software (National Institute of Health, USA, imagej.nih.gox/ij/). The gold concentration in mg/mL was quantified by measuring the signal intensity on gold K-edge images in the regions of interests (ROI). In order to compare the quantitative performances of SPCCT scans with ICP-OES (which provides the gold mass measured in the sample in µg), the gold content was estimated by multiplying the concentration by the ROI volume.

### Inductively coupled plasma-optical emission spectrometry (ICP-OES)

Animals were sacrificed at the end of the experiment, and their brains were sampled for ICP-OES analysis of gold content, according to a protocol published elsewhere 37. In brief, the harvested brain samples were homogenized before being digested in 1 mL of nitric acid at 75 °C for 24 hours. The samples were further digested for another 3 hours at 60 °C after addition of 300 µL of hydrochloric acid. 5 mL of deionized water was then added to 1 mL of the digested sample solution for ICP-OES analysis.

### Statistics

Data are presented as mean ± standard deviation. Linear regression was used to correlate gold content estimated by SPCCT analysis with that measured by ICP-OES. The agreement between gold content estimated by SPCCT analysis and by ICP-OES was assessed with Bland-Altman analysis.

## Results

For the purpose of illustration, Figure S1 shows the principle of bicolor imaging with SPCCT using a healthy rat that was intracerebrally injected with AuNP-labeled cells on one side and with gadolinium nanoparticles (GdNP)-labeled scaffold on the other side. The contrast agents could not be differentiated on conventional images, while gold K-edge images were able to detect AuNP-labeled cells in a non-equivocal manner. These GdNP were used in another SPCCT project of our consortium; 38 however, we decided to use iodinated nanoparticles instead for this study because of the artifacts GdNP produce in MRI Figure S1E).

### *In vitro* studies

**Figure 2:**
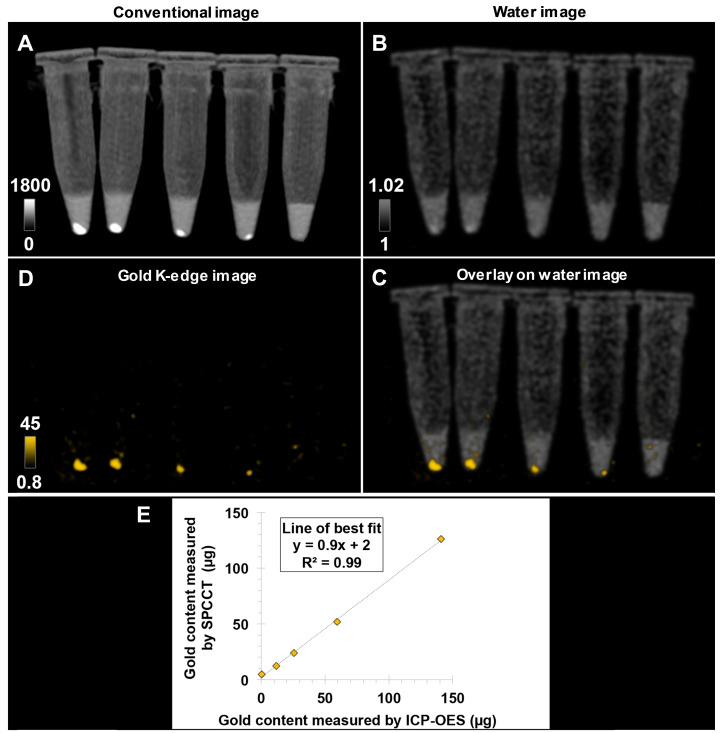
** Pellets of AuNP-labeled macrophages at decreasing cell number imaged with a single SPCCT acquisition.** (A) conventional image; (B) water image; (C) gold K-edge image; (D) overlay of gold K-edge image and water image; (E) Linear relationship between the gold content estimated by SPCCT on gold K-edge image and that measured by ICP-OES in each tube. From left to right: 1 x 10^6^, 0.5 x 10^6^, 0.25 x 10^6^, 0.125 x 10^6^ and no cells. Color bars indicate Hounsfield units for conventional images and concentration in mg/mL for material images. CT: computed tomography; ICP-OES: inductively coupled plasma-optical emission spectrometry; SPCCT: spectral photon-counting computed tomography.

Bone-marrow derived M2-polarized macrophages efficiently ingested AuNPs without any noticeable change in morphology compared to non-labeled cells in control group (Figure 1. AuNP uptake was confirmed by ICP-OES, with an average gold load per cell of 128 ± 34 pg. The cell viability was 86 ± 1% in the control group and 77 ± 3% after labeling with AuNPs. In other words, the cell viability of AuNP-labeled cells was 89% of the non-labeled cells. Both conventional and gold K-edge images of cell pellets showed an observable increase in attenuation that was dependent on the number of cells in the pellets (Figure 2). The mean volume occupied by the cells *in vitro* was 2.8 ± 1.9 µL. There was a strong linear relationship between the gold content measured on gold K-edge images by SPCCT analysis and that measured by ICP-OES in each cell pellet (R^2^=0.99). Gold content from K-edge images was slightly underestimated when compared to the measurement from ICP-OES (slope of 0.9).

### *In vivo* studies

#### Monocolor study

**Figure 3:**
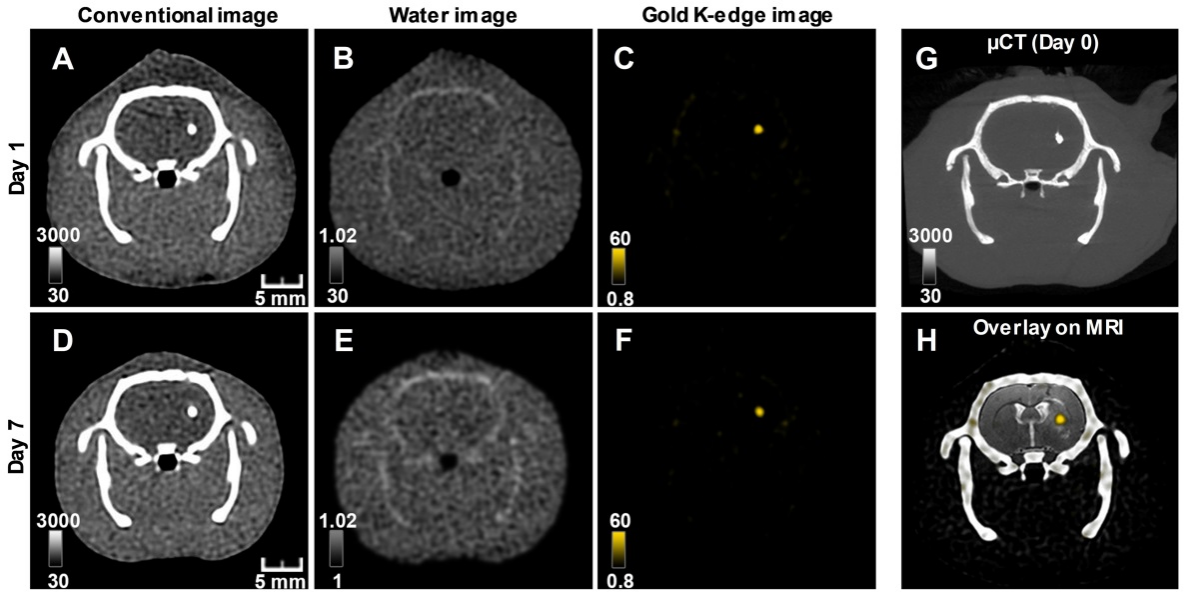
** Longitudinal SPCCT imaging of a brain-damaged rat 1 day (A-C) and 7 days (D-F) post-transplantation of 0.5 x 10^6^ AuNPs-labeled macrophages.** (A) & (D) conventional image; (B) & (E) water image; (C) & (F) gold K-edge image; (G) corresponding µCT obtained on the day of injection; (H) overlay between baseline MRI (note the striatal lesion that appears as a hyperintense signal on T2-weighted imaging), conventional and gold K-edge images. Color bars indicate Hounsfield units for conventional images and concentration in mg/mL for material images. µCT: micro-computed tomography; MRI: magnetic resonance imaging; SPCCT: spectral photon counting computed tomography.

The conventional images reconstructed from SPCCT acquisition had significantly better resolution than the CT images acquired from a (conventional) clinical scanner Figure S2. Gold K-edge images allowed a longitudinal monitoring of the brain distribution of AuNP-labeled macrophages in a non-equivocal manner (Figure 3).

**Figure 4:**
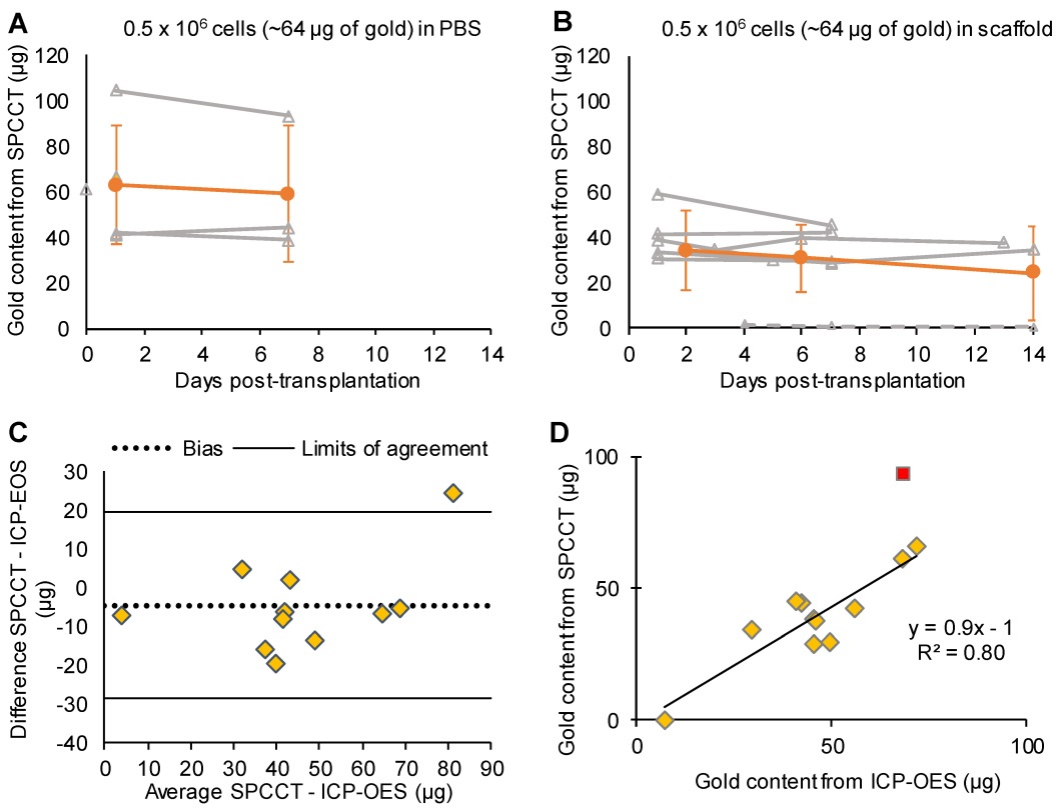
** Monocolor study: quantitative analysis.** (A) Graph plotting the average gold content estimated on gold K-edge images over time in the group of rats that received cells in PBS; (B) Graph plotting the average gold content estimated by SPCCT on gold K-edge images over time in the group of rats that received cells in scaffold; (C) Bland-Altman plot of gold content estimated by SPCCT on gold K-edge images and that measured by ICP-OES for each brain for the 2 groups; (D) Linear analysis of the same data; the red square represents an outlier that was excluded from linear regression. Grey lines represent individual animals and orange lines represent the mean ± standard deviation. Dotted line of graph B represents one animal that was excluded from analysis because of administration failure. ICP-OES: inductively coupled plasma-optical emission spectrometry; PBS: phosphate-buffered saline; SPCCT: spectral photon counting computed tomography.

One animal from the PBS group was inadvertently injected into the lateral ventricle and was therefore excluded from the quantitative analysis. This injection failure was clearly depicted by SPCCT despite cell dispersion Figure S3. Thus 12/13 animals were included in the analysis (PBS: n=5, scaffold: n=7). Moreover, one rat did not get SPCCT data at Day 14 for technical reasons. Quantitative analysis of gold content from SPCCT images revealed a heterogeneity in the gold content that was present into the brain of each rat at the first scanning time point (i.e. Day 0-3 post-injection) (Figure 4A-B). In particular, one of the rats had a very low gold signal and cell volume (1.0 µg in 0.5 µL) (Figure 4B, dashed line), possibly due to suboptimal cell administration. The gold content remained relatively stable over time in both groups that had cells delivered in PBS (Figure 4A) and in scaffold (Figure 4B). The mean volume occupied by the cells *in vivo* was 2.7 ± 1.0 µL. At the end of the experiment, the average gold content in the brain measured by ICP-OES was 48 ± 18 µg (N=12). In comparison, SPCCT analysis provided an average value of 44 ± 23 µg in the last *in vivo* imaging time point for the same 12 animals. The animal with very low gold signal had only 7.4 µg of gold in its brain as measured by ICP-OES; thus confirming the injection problem that had been detected with SPCCT.

Figure 4C shows the Bland-Altman plot describing the agreement between the gold content estimated by SPCCT analysis and that measured by ICP-OES. In average, gold K-edge images underestimated gold content by 5 µg (bias of 10%) with 95% limits of agreement of [-29; +19] µg. One animal with high gold value appeared to be an outlier (i.e. outside the limits of agreement). When excluding this animal from linear correlation analysis, the same relationship from the *in vitro* study was found between gold content from SPCCT analysis and from ICP-OES (Figure 4D, R^2^ = 0.80, slope of 0.9).

#### Bicolor study

**Figure 5:**
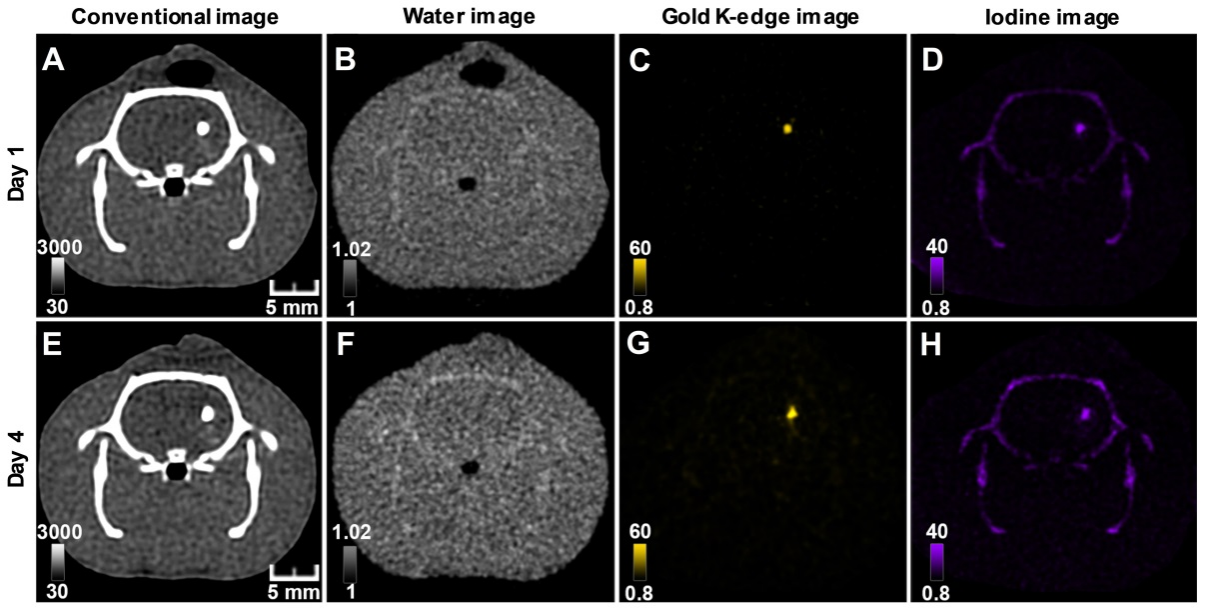
** Longitudinal SPCCT imaging of a brain-damaged rat 1 day (A-D) and 4 days (F-I) post-transplantation of 0.5 x 10^6^AuNPs-labeled macrophages embedded in INP-labeled scaffold.** (A) & (E) conventional image; (B) & (F) water image; (C) & (G) gold K-edge image; (D) & (H) iodine image. Color bars indicate Hounsfield units for conventional images and concentration in mg/mL for material images. SPCCT: spectral photon counting computed tomography; MRI: magnetic resonance imaging.

Conventional images did not allow the differentiation of AuNP-labeled cells from INP-labeled scaffold, while material images provided different gold and iodine locations, corresponding to the respective locations of cells and scaffold (Figure 5). As previously reported 28, there was some cross-talk between iodine and calcium, which resulted in appearance of the skull in the iodine image. As for monocolor imaging, bicolor imaging of gold and iodine allowed monitoring labeled cells and scaffold in the brain brain up to 2 weeks post-transplantation Figure S4. Cells and scaffold monitoring was also feasible in the mouse brain with ischemic stroke Figure S5.

**Figure 6:**
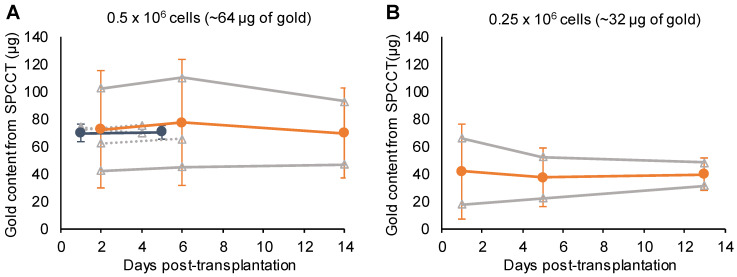
** Bicolor study: quantitative analysis.** Graph plotting the average gold content estimated on gold K-edge images over time in the group of rats that received (A) 0.5 x 10^6^; (B) 0.25 x 10^6^; and (C) 0.125 x 10^6^ cells in INP-labeled scaffold. Grey lines represent individual animals (dotted lines: 1 week follow-up and plain lines: 2 weeks follow-up); blue, respectively orange, lines represent the mean ± standard deviation of animals with 1 week, respectively 2 weeks, follow-up. SPCCT: spectral photon counting computed tomography.

One animal died at Day 12 and was imaged *ex-vivo* for the last imaging point. All animals were included in quantitative analysis (n=9). The mean volume occupied by both cells and scaffold was 4.9 ± 1.8 µL. In agreement with the monocolor study, the gold content that was present in the brain of each rat at the first scanning time point (i.e. Day 1-2 post-injection) was heterogeneous across animals and remained stable afterwards (Figure 6). The mean volume occupied by cells on gold K-edge images was 1.4 ± 0.3 µL. ICP-OES provided a value of 59 ± 8 µg of gold in the brain of rats sacrificed at Day 4 of post-transplantation (injected with 0.5 x 10^6^ cells), compared to 72 ± 4 µg estimated by SPCCT analysis at the same time point. The decrease in gold content estimated by SPCCT in each group paralleled the decrease in injected cell quantity in each group (Figure 6). The limit of detection was 4902 ± 964 AuNP-labeled cells per voxel.

## Discussion

In this study, we have shown that therapeutic cell tracking was feasible in brain-damaged rats with SPCCT. This technique enabled cell monitoring for up to 2 weeks post-injection, in a specific and quantitative manner, with a detection limit as low as 5,000 cells in a voxel of 250 × 250 × 250 µm *in vivo*. Furthermore, we have provided the proof-of-concept that simultaneous imaging with discrimination of cells and their embedding scaffold was feasible with SPCCT, thus paving the way for monitoring ATMPs combinations in the long term with the next-generation multicolor clinical SPCCT scanners.

MRI coupled with cell labeling with SPIO has been extensively used to track cells in several clinical applications including ischemic stroke 11. One major limitation of SPIO-enhanced MRI, however, is that iron oxide labeling suppresses MR signal and thus hampers the evaluation of therapeutic cells' effect on tissue regeneration by other MRI sequences. Another limitation is that cell quantification is very difficult with SPIOs. This led us to seek an alternative imaging modality, with the pre-requisite to preserve MRI contrast and allow the quantitative monitoring of cells over time. One possibility is to use perfluorocarbon contrast agents to label the cells and to image them with ^19^F MRI 39, 40. CT represents another attractive translational option, as it is widely available and intrinsically quantitative, while some radiopaque elements such as iodine and gold do not affect MR signals. Tracking therapeutic cells with CT is an emerging field (reviewed in 12). The main originality of spectral CT compared to other non-spectral X-ray-based approaches (µCT, clinical CT or synchrotron radiation CT 41) is represented by its specificity with regard to the detection of gold or other elements with K-edge energies in the appropriate range (~40 - 100 keV) 42-44. Pioneering work for the specific imaging of macrophages with SPCCT was published 10 years ago in a mouse model of atherosclerosis 45. However, the technology was not yet mature and the image acquisition took several hours, which prevented *in vivo* applications, until the development of novel SPCCT prototypes such as the one used in the current study.

Using a specific imaging approach has many advantages. First, it simplifies both data acquisition and post-processing, which is a major asset for clinical translation at the diagnostic, organizational and financial levels. Second, it allows the exclusion of confounding signals from endogenous origin due, for instance, to recent hemorrhage (which is the most common complication of ischemic stroke), intracranial calcifications (which may be found in patients especially when they are aged 46) or bone (i.e. the skull for cerebral imaging). Third, it allows differentiation of contrast agents of interest from other contrast agents. For example, iodine may be administered intravenously or intraarterially for diagnostic or interventional purposes and accumulate into the brain parenchyma as a result of blood brain barrier disruption. Alternatively, in the present study, we took advantage of this unique feature to track both cells and the cell-embedding scaffold. The capacity to discriminate two distinct contrast agents at the same location was limited to optical imaging techniques thus far. To the best of our knowledge, this is the first time that both therapeutic cells and scaffold may be tracked simultaneously in 3D and in a non-ambiguous manner using a medical imaging technique. This represents a technological leap and a great promise for evaluating ATMPs combination, for which fate and elimination rate should be evaluated independently. In this paper, our aim was to demonstrate that bicolor imaging with SPCCT can allow differentiation of therapeutic cells from their encapsulating scaffold, both labeled with distinct contrast agents. We have chosen to use gold for cell labeling and iodine for scaffold labeling in order to minimize the cross-talk that may arise from elements with closer K-edges, such as gold and gadolinium, based on previously published phantom and animal studies 17, 19, 20.

One key feature of using imaging in the context of restorative therapy is the possibility to monitor the success of cell transplantation. The variability in clinical outcome is still a clear obstacle for the adoption of cell therapies 47; however, it is crucial to determine if patients are non-responders due to a lack of treatment efficacy or to the failure of cell transplantation procedure and/or a suboptimal administration of cells. Another advantage of SPCCT is the fact that it is quantitative, thus allowing estimating the number of cells present in the sample. SPCCT provided an evaluation of gold amount that was in good agreement with that of ICP-OES taken as gold standard. This confirms and extends the quantitative performances previously obtained with different contrast agents using the same SPCCT prototype 17, 19, 38, 48. Our study demonstrates that there is an important variability in the number of transplanted cells from one animal to another, even in the highly controlled lab environment. One animal was excluded from the study because cells were injected in the lateral ventricle (which undergoes dilatation in the presence of focal cerebral injury), and one animal would have been excluded from the analysis because of the very low number of grafted cells. In the remaining animals, there was still a disparity at the baseline that could be accounted for in a therapeutic trial through a multivariate analysis. The use of SPCCT imaging coupled to cell labeling with AuNPs may thus allow a more rigorous inclusion of animals/patients and a more reliable statistical analysis of data in future preclinical and clinical trials of cell therapy. In case of intracerebral injection, using a technique that can specifically image AuNPs-labeled cells ascertains that other sources of hyperdensities, such as microhemorrhages caused by the injection procedure, are not mistakenly taken for therapeutic cells. These aspects taken together translate into a reduction of animals required for obtaining robust results in preclinical trials.

The other interest of using SPCCT imaging is in the possibility to depict the 3D cell distribution within the whole brain and to monitor their fate over a long period of time. This is essential to assess cell engraftment and to evaluate whether an early disappearance of cells might explain a lack of therapeutic efficacy. In our study, we observed very little changes in cell deployment after their injection in the brain, whether they were administered in buffer solution or embedded in a scaffold. The amount of gold that was estimated using K-edge gold imaging with SPCCT did not vary over time for a given individual. This might suggest that the number of transplanted cells remained relatively constant and that cells did not proliferate (as expected in case of macrophages). Of course, one limitation of the approach is that it does not allow determining whether cells are still labeled with AuNPs or, if this is the case, whether they are still viable and functional. However, this is true for any form of cell labeling with NPs (i.e. comparable approaches such as SPIO-enhanced or ^19^F MRI) 40, 49. Indeed, AuNPs may be released from cells and taken up by nearby endogenous macrophages. We were not able to evaluate this hypothesis in our study because we used repair macrophages as therapeutic cells. We have chosen these cells both because they represent promising candidates to treat ischemic stroke patients 24, 25 and because they are easy to label with NPs due to their phagocytic capacities 50, 51. Future studies should aim at discriminating injected cells from endogenous ones by making use of cells with bioluminescence/fluorescence or by revealing specific epitopes for instance for cells of human origin. We did not see any elimination of AuNPs during the course of the study, which demonstrates the feasibility of long-term tracking but may also bring up a safety concern. It would be interesting to conduct a longitudinal imaging study over longer periods of time to define the time course (and pathways) of gold elimination from the brain.

One limitation of X-ray based techniques for cellular imaging is their intrinsic low sensitivity. Nevertheless, it is interesting to note that the detection limit of SPCCT imaging was of the same order of magnitude as that of ^19^F MRI (i.e. 10^3^-10^4^ cells per voxel). This initial study used conventional reconstruction by filtered back-projection therefore there is scope for improvement of sensitivity, for example by implementation of iterative reconstruction 52, 53. In addition, AuNP load per cell may be further optimized 54. In our study, the detection limits have been basically determined for informative purposes and should be investigated more thoroughly as a next step. However, the main difficulty is the need for a gold standard technique that would allow precise evaluation of the gold amount in a given voxel. To address this issue, most brains of the current study have been further imaged with *in vivo* K-edge imaging and ex vivo phase-contrast imaging using X-rays from Synchrotron radiations (data analysis is on-going and will be published elsewhere). Another limitation of SPCCT for cell tracking may be patient exposure to ionizing radiations. Radiotoxicity is not an issue for longitudinal follow-up of rodents with µCT 55, 56. In the same way, SPCCT may allow safe, multiple exams of the same patient since the scans are performed at a much lower dose when compared to conventional CT for an equivalent image quality and also because of the elimination for the need of prescan 14, 48, 57-60.

The SPCCT prototype that we have used for this study displayed all the characteristics of a clinical CT scanner, but a reduced field of view 28. Due to improved image quality compared to conventional CT, we were able to image rats and mice brains with sufficient details to track therapeutic cells. SPCCT clinical scanners with full field of view are now available 61, including in our own institution. Therefore, there is hope that the SPCCT approach that we propose may help investigations of cell therapy and ATMP combinations in future clinical trials. Some hurdles, however, need to be overcome in the preclinical field beforehand. The main questions arise from the fate of contrast agents in the body (e.g. potential accumulation in the brain) and the possible interactions between the contrast agents and the therapeutic effects. It would be too long to debate these issues here, but two points might be interesting to consider with regard to the use of gold as the cell label. First, it has been shown recently that macrophages were able to scavenge AuNPs and eliminate them through exosomes 62. Second, previous studies have shown that macrophage function was not affected by gold loading 29, 63. Of course, these issues should be addressed in well-designed *in vitro* and *in vivo* experiments. We believe that our results represent a major milestone from a methodological point of view and should facilitate better characterization and optimization of cell therapies at the preclinical level. In the present study, rats were not submitted to stroke because our main purpose was to evaluate a novel imaging approach; thus a 'morphological' and 'radiological' model of brain lesion that did not induce mortality. One of the perspectives of our work is to evaluate the effects of the intracerebral administration of therapeutic cells aimed at modulating neuroinflammation (such as M2-polarized macrophages or human mesenchymal stem cells) in a rat model of ischemic stroke by coupling multimodal imaging (SPCCT for monitoring cells and scaffold and advanced multiparametric MRI methods for evaluating tissue repair) with neurobehavior and immunohistochemistry analyses. Because of its translational nature, this protocol may help bridging the gap between preclinical and clinical trials, and thus foster the future approval of efficient cell therapies and ATMP combinations for stroke patients.

## Conclusions

Multicolor CT is an innovative translational imaging tool that allows monitoring and quantifying therapeutic cells and their encapsulating scaffold transplanted into the damaged rat brain.

## Figures and Tables

**Table 1:** System characteristics and acquisition parameters for each imaging modality.

	Computed tomography (CT)		
	µCT	CT	SPCCT
**System**	Siemens INVEON	GE Brightspeed	Philips prototype
**Tension (kVp)**	80	120	120
**Current (mA)**	500	150	100
**In-plane resolution (µm)**	56	310	250
**Slice thickness (µm)**	56	625	250
**Energy thresholds (keV)**	N/A	N/A	30, 53, 78, 83, 98
**Acquisition time (sec)**	792	3	17
	**Magnetic resonance imaging (MRI)**		
**System**	Bruker Avance II	**Sequence**	T2 - RARE factor 8
**Field strength**	7T	**TE/TR (ms/ms)**	57.7/5000 ms
**Gradients**	440 mT/m	**In-plane resolution (µm)**	117
**Software**	ParaVision 5.1	**Slice thickness (µm)**	800
**Coils diameters (mm)**	15 / 25	**Acquisition time (min)**	4

µCT: micro-computed tomography; RARE: rapid acquisition with relaxation enhancement; SPCCT: spectral photon counting computed tomography; TE: echo time; TR: repetition time.

## Abbreviations

11-MUDA: 11-mercaptoundecanoic acid
3D: three-dimensional
ATMPs: advanced-therapy medicinal products
AuNPs: gold nanoparticles
CT: computed tomography
ICP-OES: inductively coupled plasma-optical emission spectrometry
INPs: iodinated nanoparticles
LPS: lipopolysaccharide
µCT: micro-computed tomography
mM-CSF: mouse macrophage-colony stimulating factor
MRI: magnetic resonance imaging
PBS: phosphate-buffered saline
PDI: polydispersity index
pMCAO: permanent middle cerebral artery occlusion
RPMI: Roswell Park Memorial Institute
SPCCT: spectral photon counting computed tomography
SPIO: superparamagnetic particles of iron oxide
THF: tetrahydrofuran
TIB-PVAL: 2,3,5-triodobenzoyl ester of poly(vinyl alcohol)
UV: ultraviolet

## References

[1] Detante O, Moisan A, Hommel M, Jaillard A (2017). Controlled clinical trials of cell therapy in stroke: Meta-analysis at six months after treatment. Int J Stroke.

[2] Lees JS, Sena ES, Egan KJ, Antonic A, Koblar SA, Howells DW (2012). Stem cell-based therapy for experimental stroke: a systematic review and meta-analysis. Int J Stroke.

[3] Wei L, Wei ZZ, Jiang MQ, Mohamad O, Yu SP (2017). Stem cell transplantation therapy for multifaceted therapeutic benefits after stroke. Prog Neurobiol.

[4] Chen L, Zhang G, Khan AA, Guo X, Gu Y (2016). Clinical Efficacy and Meta-Analysis of Stem Cell Therapies for Patients with Brain Ischemia. Stem Cells Int.

[5] Steinberg GK, Kondziolka D, Wechsler LR, Lunsford LD, Coburn ML, Billigen JB (2016). Clinical Outcomes of Transplanted Modified Bone Marrow-Derived Mesenchymal Stem Cells in Stroke: A Phase 1/2a Study. Stroke.

[6] Kalladka D, Sinden J, Pollock K, Haig C, McLean J, Smith W (2016). Human neural stem cells in patients with chronic ischaemic stroke (PISCES): a phase 1, first-in-man study. Lancet.

[7] Boisserand LS, Kodama T, Papassin J, Auzely R, Moisan A, Rome C (2016). Biomaterial Applications in Cell-Based Therapy in Experimental Stroke. Stem Cells Int.

[8] Modo M, Kolosnjaj-Tabi J, Nicholls F, Ling W, Wilhelm C, Debarge O (2013). Considerations for the clinical use of contrast agents for cellular MRI in regenerative medicine. Contrast media & molecular imaging.

[9] McColgan P, Sharma P, Bentley P (2011). Stem cell tracking in human trials: a meta-regression. Stem Cell Rev.

[10] Shaikh FA, Kurtys E, Kubassova O, Roettger D (2020). Reporter gene imaging and its role in imaging-based drug development. Drug discovery today.

[11] Bernsen MR, Guenoun J, van Tiel ST, Krestin GP (2015). Nanoparticles and clinically applicable cell tracking. The British journal of radiology.

[12] Kim J, Chhour P, Hsu J, Litt HI, Ferrari VA, Popovtzer R (2017). Use of Nanoparticle Contrast Agents for Cell Tracking with Computed Tomography. Bioconjugate chemistry.

[13] Yeh BM, FitzGerald PF, Edic PM, Lambert JW, Colborn RE, Marino ME (2017). Opportunities for new CT contrast agents to maximize the diagnostic potential of emerging spectral CT technologies. Advanced drug delivery reviews.

[14] Taguchi K, Iwanczyk JS (2013). Vision 20/20: Single photon counting x-ray detectors in medical imaging. Medical physics.

[15] Sigovan M, Si-Mohamed S, Bar-Ness D, Mitchell J, Langlois JB, Coulon P (2019). Feasibility of improving vascular imaging in the presence of metallic stents using spectral photon counting CT and K-edge imaging. Scientific reports.

[16] Kopp FK, Daerr H, Si-Mohamed S, Sauter AP, Ehn S, Fingerle AA (2018). Evaluation of a preclinical photon-counting CT prototype for pulmonary imaging. Scientific reports.

[17] Si-Mohamed S, Bar-Ness D, Sigovan M, Tatard-Leitman V, Cormode DP, Naha PC (2018). Multicolour imaging with spectral photon-counting CT: a phantom study. Eur Radiol Exp.

[18] Muenzel D, Bar-Ness D, Roessl E, Blevis I, Bartels M, Fingerle AA Spectral Photon-counting CT: Initial Experience with Dual-Contrast Agent K-Edge Colonography. Radiology. 2016:160890.

[19] Cormode DP, Si-Mohamed S, Bar-Ness D, Sigovan M, Naha PC, Balegamire J (2017). Multicolor spectral photon-counting computed tomography: *in vivo* dual contrast imaging with a high count rate scanner. Scientific reports.

[20] Dangelmaier J, Bar-Ness D, Daerr H, Muenzel D, Si-Mohamed S, Ehn S (2018). Experimental feasibility of spectral photon-counting computed tomography with two contrast agents for the detection of endoleaks following endovascular aortic repair. Eur Radiol.

[21] Riederer I, Si-Mohamed S, Ehn S, Bar-Ness D, Noel PB, Fingerle AA (2019). Differentiation between blood and iodine in a bovine brain-Initial experience with Spectral Photon-Counting Computed Tomography (SPCCT). PloS one.

[22] Si-Mohamed S, Thivolet A, Bonnot PE, Bar-Ness D, Kepenekian V, Cormode DP (2018). Improved Peritoneal Cavity and Abdominal Organ Imaging Using a Biphasic Contrast Agent Protocol and Spectral Photon Counting Computed Tomography K-Edge Imaging. Investigative radiology.

[23] Si-Mohamed S, Tatard-Leitman V, Laugerette A, Sigovan M, Pfeiffer D, Rummeny EJ (2019). Spectral Photon-Counting Computed Tomography (SPCCT): in-vivo single-acquisition multi-phase liver imaging with a dual contrast agent protocol. Scientific reports.

[24] Chernykh ER, Kafanova MY, Shevela EY, Sirota SI, Adonina EI, Sakhno LV (2014). Clinical experience with autologous M2 macrophages in children with severe cerebral palsy. Cell Transplant.

[25] Chernykh ER, Shevela EY, Starostina NM, Morozov SA, Davydova MN, Menyaeva EV (2016). Safety and therapeutic potential of M2-macrophages in stroke treatment. Cell Transplant.

[26] Leung GK, Wang YC, Wu W (2012). Peptide nanofiber scaffold for brain tissue reconstruction. Methods Enzymol.

[27] Zhang N, Luo Y, He L, Zhou L, Wu W (2016). A self-assembly peptide nanofibrous scaffold reduces inflammatory response and promotes functional recovery in a mouse model of intracerebral hemorrhage. Nanomedicine.

[28] Si-Mohamed S, Bar-Ness D, Sigovan M, Cormode D, Coulon P, Coche E (2017). Review of an initial experience with an experimental spectral photon-counting computed tomography system. Nuclear Instruments and Methods in Physics Research.

[29] Chhour P, Naha PC, O'Neill SM, Litt HI, Reilly MP, Ferrari VA (2016). Labeling monocytes with gold nanoparticles to track their recruitment in atherosclerosis with computed tomography. Biomaterials.

[30] Chhour P, Naha PC, Cheheltani R, Benardo B, Mian S, Cormode DP (2016). Gold Nanoparticles for Biomedical Applications: Synthesis and *In vitro* Evaluation. In: Lu ZR SS, editor. Nanomaterials in Pharmacology Methods in Pharmacology and Toxicology. New York: Humana Press.

[31] Balegamire J, Vandamme M, Chereul E, Si-Mohamed S, Azzouz Maache S, Almouazen E (2020). Iodinated polymer nanoparticles as contrast agent for Spectral Photon Counting Computed Tomography. submitted.

[32] Kaneko A, Matsushita A, Sankai Y (2015). A 3D nanofibrous hydrogel and collagen sponge scaffold promotes locomotor functional recovery, spinal repair, and neuronal regeneration after complete transection of the spinal cord in adult rats. Biomed Mater.

[33] Sridharan S, Lepelletier FX, Trigg W, Banister S, Reekie T, Kassiou M (2017). Comparative Evaluation of Three TSPO PET Radiotracers in a LPS-Induced Model of Mild Neuroinflammation in Rats. Mol Imaging Biol.

[34] Llovera G, Roth S, Plesnila N, Veltkamp R, Liesz A Modeling stroke in mice: permanent coagulation of the distal middle cerebral artery. Journal of visualized experiments: JoVE. 2014(89e): 51729.

[35] Roessl E, Proksa R (2007). K-edge imaging in x-ray computed tomography using multi-bin photon counting detectors. Physics in medicine and biology.

[36] Hubbell JH, Seltzer SM (2004). Tables of x-ray mass attenuation coefficients and mass energy-absorption coefficients. In: National Institute of Standards and Technology G, editor.

[37] Naha PC, Lau KC, Hsu JC, Hajfathalian M, Mian S, Chhour P (2016). Gold silver alloy nanoparticles (GSAN): an imaging probe for breast cancer screening with dual-energy mammography or computed tomography. Nanoscale.

[38] Halttunen N, Lerouge F, Chaput F, Vandamme M, Karpati S, Si-Mohamed S (2019). Hybrid Nano-GdF3 contrast media allows pre-clinical *in vivo* element-specific K-edge imaging and quantification. Scientific reports.

[39] Bible E, Dell'Acqua F, Solanky B, Balducci A, Crapo PM, Badylak SF (2012). Non-invasive imaging of transplanted human neural stem cells and ECM scaffold remodeling in the stroke-damaged rat brain by (19)F- and diffusion-MRI. Biomaterials.

[40] Khurana A, Chapelin F, Xu H, Acevedo JR, Molinolo A, Nguyen Q (2018). Visualization of macrophage recruitment in head and neck carcinoma model using fluorine-19 magnetic resonance imaging. Magn Reson Med.

[41] Astolfo A, Arfelli F, Schultke E, James S, Mancini L, Menk RH (2013). A detailed study of gold-nanoparticle loaded cells using X-ray based techniques for cell-tracking applications with single-cell sensitivity. Nanoscale.

[42] Kim J, Bar-Ness D, Si-Mohamed S, Coulon P, Blevis I, Douek P (2018). Assessment of candidate elements for development of spectral photon-counting CT specific contrast agents. Scientific reports.

[43] Dong YC, Hajfathalian M, Maidment PSN, Hsu JC, Naha PC, Si-Mohamed S (2019). Effect of Gold Nanoparticle Size on Their Properties as Contrast Agents for Computed Tomography. Scientific reports.

[44] Pan D, Schirra CO, Senpan A, Schmieder AH, Stacy AJ, Roessl E (2012). An early investigation of ytterbium nanocolloids for selective and quantitative "multicolor" spectral CT imaging. ACS nano.

[45] Cormode DP, Roessl E, Thran A, Skajaa T, Gordon RE, Schlomka JP (2010). Atherosclerotic plaque composition: analysis with multicolor CT and targeted gold nanoparticles. Radiology.

[46] Yalcin A, Ceylan M, Bayraktutan OF, Sonkaya AR, Yuce I (2016). Age and gender related prevalence of intracranial calcifications in CT imaging; data from 12,000 healthy subjects. Journal of chemical neuroanatomy.

[47] Meir R, Motiei M, Popovtzer R (2014). Gold nanoparticles for *in vivo* cell tracking. Nanomedicine (London, England).

[48] Si-Mohamed S, Cormode DP, Bar-Ness D, Sigovan M, Naha PC, Langlois JB (2017). Evaluation of spectral photon counting computed tomography K-edge imaging for determination of gold nanoparticle biodistribution *in vivo*. Nanoscale.

[49] Meir R, Popovtzer R (2018). Cell tracking using gold nanoparticles and computed tomography imaging. WIRES Nanomed Nanobiotechnol.

[50] Riou A, Chauveau F, Cho TH, Marinescu M, Nataf S, Nighoghossian N (2013). MRI assessment of the intra-carotid route for macrophage delivery after transient cerebral ischemia. NMR Biomed.

[51] MacParland SA, Tsoi KM, Ouyang B, Ma XZ, Manuel J, Fawaz A (2017). Phenotype Determines Nanoparticle Uptake by Human Macrophages from Liver and Blood. ACS nano.

[52] Bernstein AL, Dhanantwari A, Jurcova M, Cheheltani R, Naha PC, Ivanc T (2016). Improved sensitivity of computed tomography towards iodine and gold nanoparticle contrast agents via iterative reconstruction methods. Scientific reports.

[53] Mory C, Sixou B, Si-Mohamed S, Boussel L, Rit S (2018). Comparison of five one-step reconstruction algorithms for spectral CT. Physics in medicine and biology.

[54] Betzer O, Meir R, Dreifuss T, Shamalov K, Motiei M, Shwartz A (2015). In-vitro Optimization of Nanoparticle-Cell Labeling Protocols for In-vivo Cell Tracking Applications. Scientific reports.

[55] Vande Velde G, De Langhe E, Poelmans J, Bruyndonckx P, d'Agostino E, Verbeken E (2015). Longitudinal *in vivo* microcomputed tomography of mouse lungs: No evidence for radiotoxicity. Am J Physiol Lung Cell Mol Physiol.

[56] Berghen N, Dekoster K, Marien E, Dabin J, Hillen A, Wouters J (2019). Radiosafe micro-computed tomography for longitudinal evaluation of murine disease models. Scientific reports.

[57] McCollough CH, Chen GH, Kalender W, Leng S, Samei E, Taguchi K (2012). Achieving routine submillisievert CT scanning: report from the summit on management of radiation dose in CT. Radiology.

[58] Yu Z, Leng S, Li Z, Halaweish AF, Kappler S, Ritman EL (2016). How Low Can We Go in Radiation Dose for the Data-Completion Scan on a Research Whole-Body Photon-Counting Computed Tomography System. J Comput Assist Tomogr.

[59] Klein L, Dorn S, Amato C, Heinze S, Uhrig M, Schlemmer HP (2020). Effects of Detector Sampling on Noise Reduction in Clinical Photon-Counting Whole-Body Computed Tomography. Investigative radiology.

[60] Pourmorteza A, Symons R, Henning A, Ulzheimer S, Bluemke DA (2018). Dose Efficiency of Quarter-Millimeter Photon-Counting Computed Tomography: First-in-Human Results. Investigative radiology.

[61] Symons R, Pourmorteza A, Sandfort V, Ahlman MA, Cropper T, Mallek M (2017). Feasibility of Dose-reduced Chest CT with Photon-counting Detectors: Initial Results in Humans. Radiology.

[62] Logozzi M, Mizzoni D, Bocca B, Di Raimo R, Petrucci F, Caimi S (2019). Human primary macrophages scavenge AuNPs and eliminate it through exosomes. A natural shuttling for nanomaterials. European journal of pharmaceutics and biopharmaceutics: official journal of Arbeitsgemeinschaft fur Pharmazeutische Verfahrenstechnik eV.

[63] Domey J, Teichgraber U, Hilger I (2015). Gold nanoparticles allow detection of early-stage edema in mice via computed tomography imaging. International journal of nanomedicine.

