# Supplementary material for: Multicolor spectral photon counting CT monitors and quantifies therapeutic cells and their encapsulating scaffold in a model of brain damage
Source: Nanotheranostics. 2020 Apr 22;4(3):129–41. doi: 10.7150/ntno.45354 (PMC7256015; doi:10.7150/ntno.45354)
Supplement: Supplementary file 1 — Supplementary figures. [file ntnov04p0129s1.pdf]

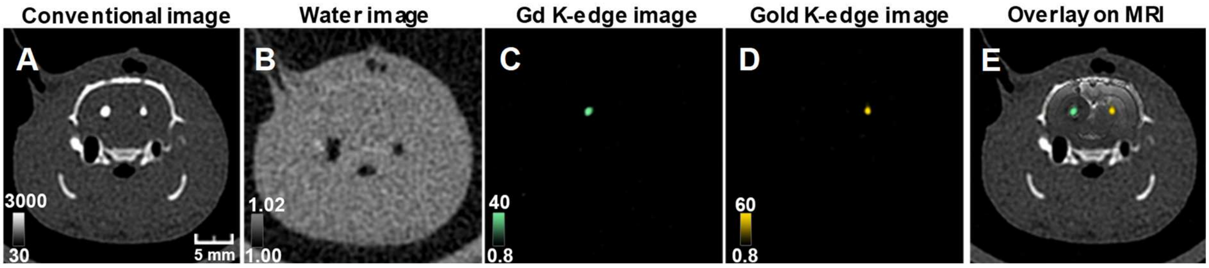

Figure S1. Principle of bicolor imaging with a single SPCCT acquisition demonstrated in a healthy rat intracerebrally injected with GdNP-labeled scaffold in the left hemisphere and AuNP-labeled cells in the right hemisphere. (A) conventional image; (B) water (non-contrast) image; (C) Gadolinium (Gd) K-edge image; (D) Gold K-edge image and (E) overlay between MRI, conventional, gold and gadolinium K-edge images. Note the artifact produced by gadolinium (hypointense signal on T_2-weighted MRI) while gold does not impact the MR signal: in subsequent studies, we used iodine (which does not impact the MR signal either) to label the scaffold rather than gadolinium to preserve MR signals for the longitudinal evaluation of brain repair with multiparametric MRI. Colorbars indicate Hounsfield units for conventional images and concentration in mg/mL for material images. MRI: magnetic resonance imaging; SPCCT: spectral photon counting computed tomography.

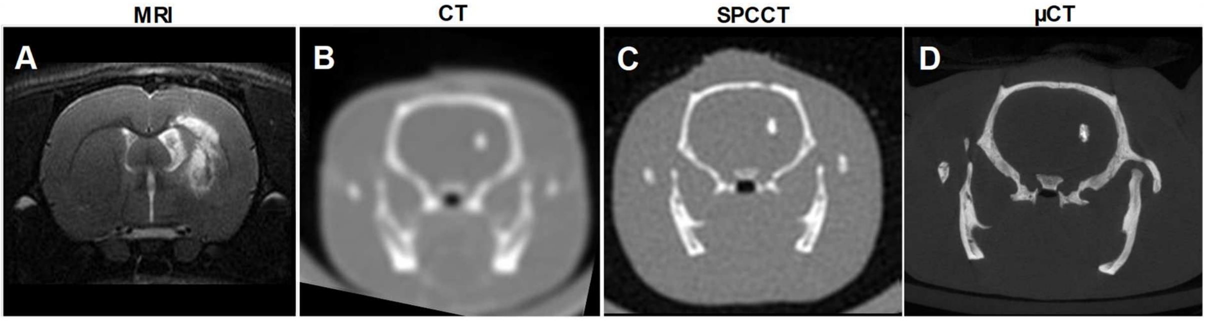

Figure S2. Multimodal imaging of a brain-damaged rat at 1 day post-transplantation of 0.5 x 10^6 AuNPs-labeled macrophages. (A) baseline MRI: the lesion is seen as a hyperintense signal in the striatum on T2-weighted MRI – also note the dilatation of the ipsilateral lateral ventricle; (B) conventional image obtained with CT (voxel size: 310 × 310 × 625 µm); (C) conventional image obtained with SPCCT (voxel size: 250 × 250 × 250 µm) and (D) conventional image obtained with µCT (voxel size: 56 × 56 × 56 µm). CT: computed tomography; µCT: micro-computed tomography; MRI: magnetic resonance imaging; SPCCT: spectral photon counting computed tomography.

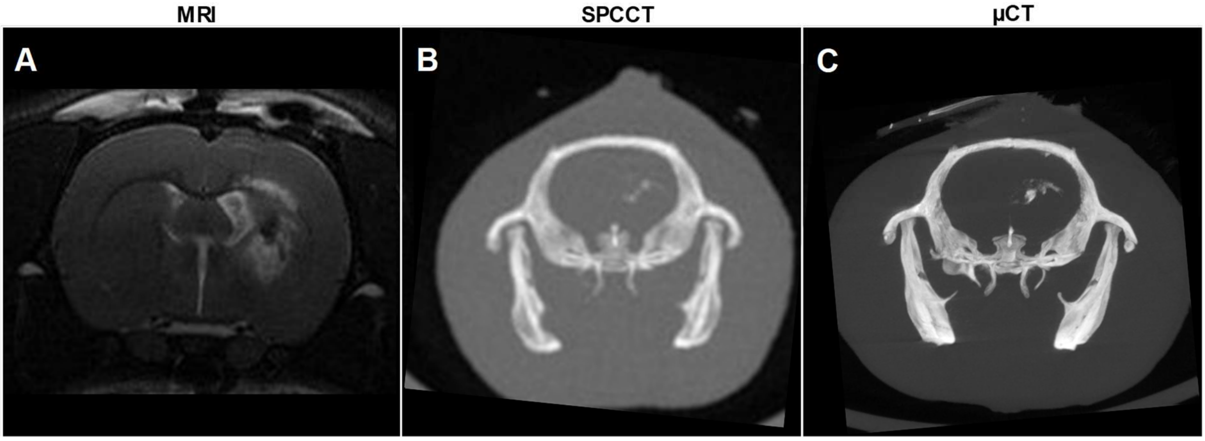

Figure S3. Multimodal imaging of a brain-damaged rat at 1 day post-transplantation of 0.5 x 10^6AuNPs-labeled macrophages that was inadvertently injected into the ipsilateral lateral ventricle. (A) baseline MRI: the lesion is seen as a hyperintense signal in the striatum on T2-weighted MRI – note the hypointense signal within the lesion indicative of hemorrhage and the dilatation of the ipsilateral lateral ventricle; (B) conventional image obtained with SPCCT: the administration failure is clearly seen even if cells were dispersed; (C) conventional image obtained with µCT. µCT: micro-computed tomography; MRI: magnetic resonance imaging; SPCCT: spectral photon counting computed tomography.

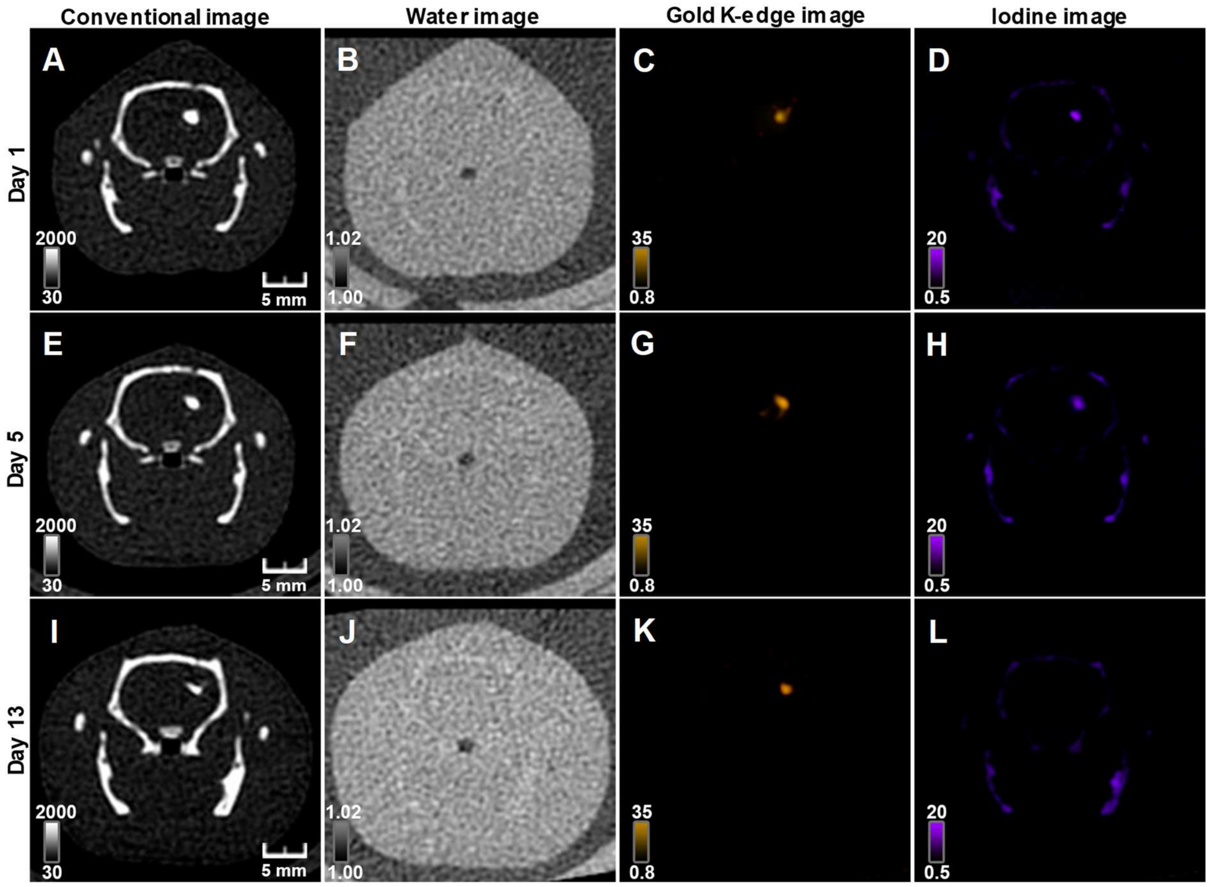

Figure S4. Longitudinal SPCCT imaging of a brain-damaged rat 1 day (A-D), 5 days (E-H) and 13 days (I-L) post-transplantation of 0.25 x 10^6 AuNPs-labeled macrophages embedded in INP-labeled scaffold. (A), (E) & (I) conventional image; (B), (F) & (J) water image; (C), (G) & (K) gold K-edge image; (D), (H) & (L) iodine image. Color bars indicate Hounsfield units for conventional images and concentration in mg/mL for material images. SPCCT: spectral photon counting computed tomography.

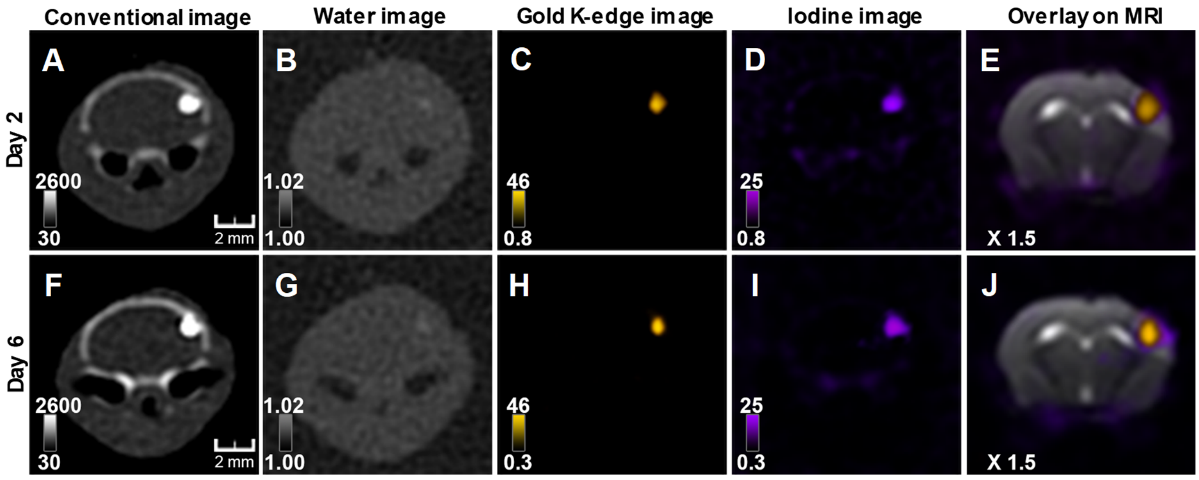

Figure S5. Longitudinal SPCCT imaging of a stroke-induced mouse at 2 days (A-E) and 6 days (F-J) post-transplantation of 0.5 x 10^6 AuNPs-labeled macrophages in INPs-labeled scaffold. (A) & (F) conventional images; (B) & (G) water images; (C) & (H) gold K-edge images; (D) & (I) iodine images and (E) & (J) overlay between MRI, gold K-edge and iodine images. Note the cortical ischemic lesion seen as a hyperintense signal on T2-weighted imaging. Color bars indicate Hounsfield units for conventional images and concentration in mg/mL for material images. MRI: magnetic resonance imaging; SPCCT: spectral photon counting computed tomography.
